# Comparative transcriptome and proteome analysis of resistant and susceptible *Talaromyces marneffei* clinical isolates reveals a systemic multitiered defense network against azoles

**DOI:** 10.1128/aac.00293-26

**Published:** 2026-09-02

**Authors:** Zheng Yanqing, Peng Gaoyuan, Pang Xiaofeng, Liang Chaojuan, Luo Xiaolu, Wei Cailing, Qin Jianglong, Zou Jun, Wang Zhiyi, Pan Kaisu, Du Mingwei, Xue Peng, Liao Wanqing, Yu Mei

**Affiliations:** 1 The Fourth People`s Hospital of Nanning, Nanning, China; 2 Guangxi Key Laboratory of Mycosis Prevention and Treatment, Department of Dermatology and Venereology, The First Affiliated Hospital of Guangxi Medical University 117742, Nanning, China; 3 Department of Dermatology, the Second Affiliated Hospital, Naval Medical University 12521 , Shanghai, China; 4 Nantong Key Laboratory of Environmental Toxicology, Department of Occupational Medicine and Environmental Toxicology, School of Public Health, Nantong University 66479, Nantong, China; University Children's Hospital Münster, Münster, Germany

**Keywords:** *Talaromyces marneffei*, azole resistance, transcriptomics, proteomics, multi-omics, biomarkers

## Abstract

*Talaromyces marneffei* is a thermally dimorphic fungus causing life-threatening systemic mycosis in immunocompromised patients. The emergence of azole-resistant clinical isolates, especially those with reduced susceptibility to voriconazole (VOC) and fluconazole (FLC), presents a critical therapeutic challenge. Although target-gene alterations and efflux-related mechanisms have been reported in pathogenic fungi, the broader molecular basis distinguishing high-minimum-inhibitory concentration (MIC) and low-MIC *T. marneffei* clinical isolates remains incompletely defined. Here, we performed integrated transcriptomic (RNA-seq), proteomic (DIA-LC-MS/MS), and RT-qPCR analyses comparing four resistant and four susceptible *T. marneffei* clinical isolates. Differentially expressed genes and proteins were identified, functionally interpreted, and integrated to prioritize resistance-associated molecular candidates. The multi-omics results supported a constitutive, multitiered resistance-associated state involving transport and cell wall processes, redox-detoxification, mitochondrial metabolism, and stress regulatory adaptation. Signals involving coilin, CatA, and other discriminant features contributed to the multi-omics prioritization layer. RT-qPCR supported a 17-gene panel associated with transport, redox-detoxification, mitochondrial metabolism, and stress regulation. Five genes, PMAA_008970, PMAA_058960, PMAA_070510, PMAA_088690, and PMAA_092180, showed statistically significant R/S differences, while the remaining panel members were detectable and trend-supporting within the same functional framework. These findings refine a hierarchical azole-resistance model in *T. marneffei* and provide a focused candidate panel for future mechanistic validation.

## INTRODUCTION

*Talaromyces marneffei (T. marneffei*), formerly known as *Penicillium marneffei*, is a thermally dimorphic fungus that causes talaromycosis, a life-threatening systemic mycosis primarily affecting human immunodeficiency virus (HIV)-infected patients and other immunocompromised individuals in Southeast Asia and Southern China (1–3). As a clinically significant pathogen, *T. marneffei* poses a severe challenge to public health due to its high morbidity and mortality rates, which can reach up to 40% in untreated or delayed cases (4, 5). Current treatment regimens include induction therapy with amphotericin B followed by consolidation and maintenance therapy with azoles, particularly itraconazole (2, 6). Voriconazole (VOC) is an important alternative for patients who cannot tolerate amphotericin B and shows potent activity in susceptibility testing and clinical settings (6, 7). Fluconazole (FLC) is occasionally used for prophylaxis in resource-limited settings (6), although its utility is limited by higher minimum-inhibitory concentrations (MICs) compared with other azoles in susceptibility studies (7–11). With increasing clinical exposure to azoles, resistant *T. marneffei* isolates have become an important therapeutic concern. In clinical studies, elevated FLC MICs and alterations in resistance-associated genes such as *MDR1* and *Cyp51B* have been associated with delayed blood-culture clearance after antifungal therapy (4, 12).

Resistance to azoles in pathogenic fungi is a complex phenotype involving efflux-pump activation, alteration of the target enzyme lanosterol 14α-demethylase, stress-response remodeling, and broader metabolic adaptation (13, 14). Although these mechanisms have been extensively characterized in model fungal pathogens such as *Candida albicans* and *Aspergillus fumigatus* (13, 15), the molecular features that distinguish resistant and susceptible *T. marneffei* clinical isolates remain insufficiently defined. Clinical and laboratory studies show substantial diversity in *T. marneffei* antifungal susceptibility, including elevated FLC MICs and variable responses to VOC among different isolates (8–11). Defining the transcriptomic and proteomic basis of these phenotypes is, therefore, essential for understanding azole-response stratification and identifying resistance-associated molecular markers.

In recent years, the integration of high-throughput omics technologies, particularly transcriptomics and proteomics, has provided a comprehensive platform for elucidating the complex molecular networks underlying biological phenotypes (16, 17). Transcriptome sequencing (RNA-seq) quantifies gene expression on a global scale, whereas proteomics complements transcriptome data by capturing protein-level effectors and post-translational regulation (18–20). In *T. marneffei*, genomic, transcriptomic, proteomic, and metabolomic studies have revealed molecular programs associated with environmental adaptation, dimorphic transition, host-cell interaction, and antifungal responses (21–24). However, integrated transcriptome-proteome profiling directly comparing high-MIC and low-MIC *T. marneffei* clinical isolates remains limited.

In this study, we performed a comparative analysis of the transcriptome and proteome profiles of eight *T. marneffei* clinical isolates, comprising four resistant strains (exhibiting high MICs to VOC and FLC) and four susceptible strains. By integrating transcriptomic and proteomic data, we aimed to identify key differentially expressed genes (DEGs) and differentially expressed proteins (DEPs) associated with azole resistance. Furthermore, we sought to uncover the critical metabolic pathways and regulatory networks that confer the resistant phenotype. These findings align with previous observations that azole resistance in dimorphic fungi like *T. marneffei* often involves efflux pump overexpression and alterations in sterol biosynthesis pathways (10, 12). The integration of multi-omics approaches has proven effective in elucidating similar resistance mechanisms in related fungal pathogens (24). The findings of this study provide a theoretical basis for elucidating the mechanisms of drug resistance in *T. marneffei*, including potential cross-resistance between VOC and FLC (10), and offer potential biomarkers for the rapid diagnosis and targeted treatment of resistant infections.

## MATERIALS AND METHODS

**Fig 1:**
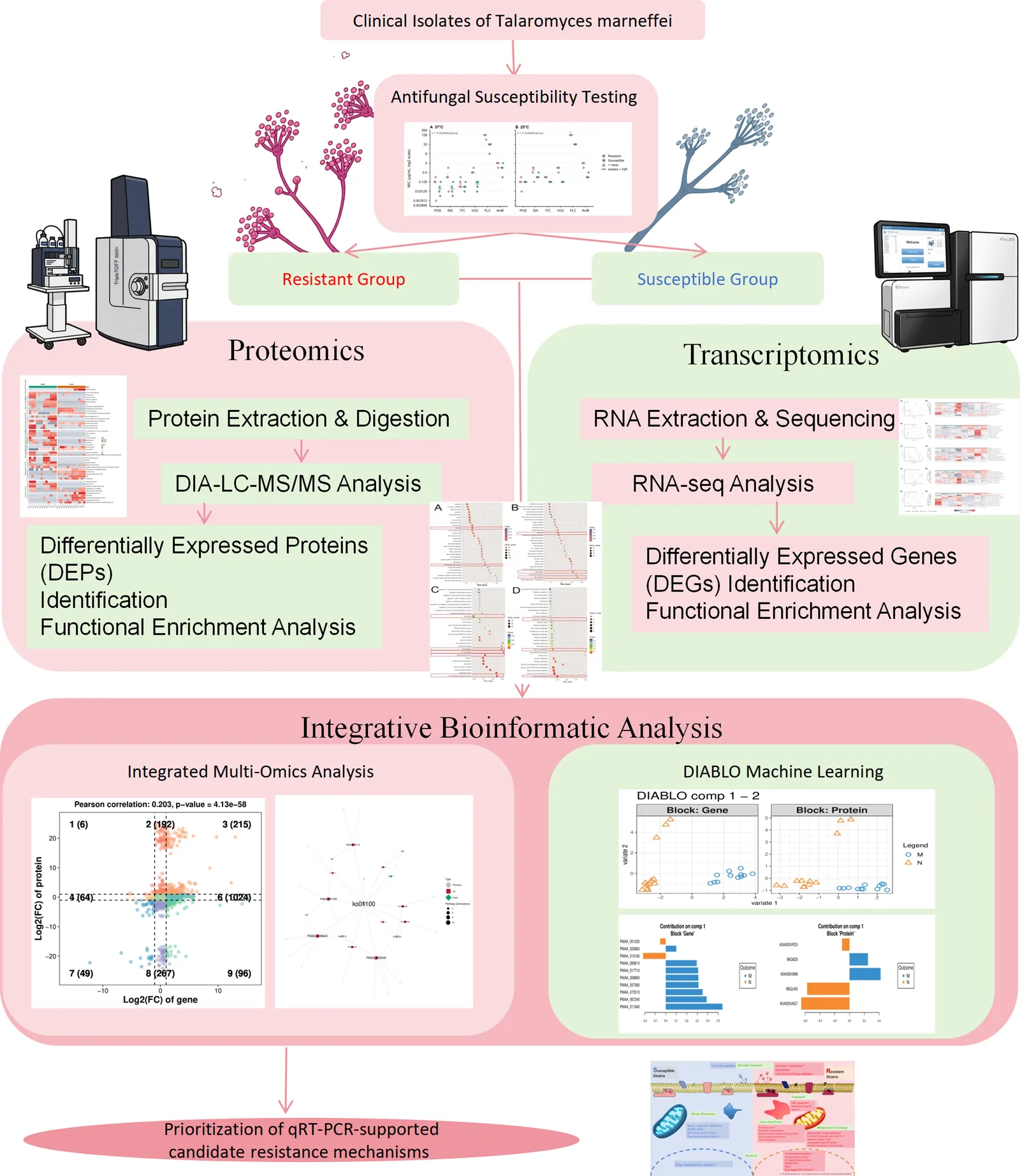
Schematic workflow of the integrated multi-omics analysis. Clinical isolates of *T. marneffei* were stratified into resistant and susceptible groups based on antifungal susceptibility profiles and morphology-supported identification. Transcriptomic, proteomic, enrichment, multi-omics integration, DIABLO prioritization, and RT-qPCR validation layers were combined to prioritize resistance-associated molecular candidates and generate a qRT-PCR-supported candidate resistance model.

**Fig 2:**
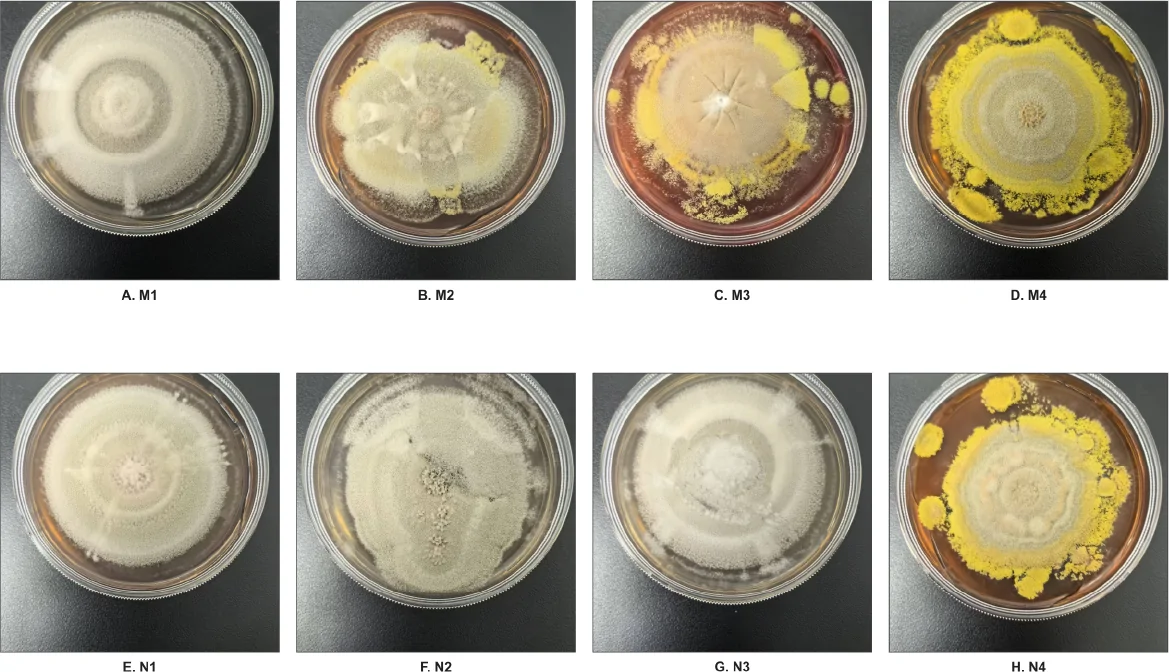
Mycelial-phase colony morphology of *T. marneffei* clinical isolates cultured at 25°C. Representative colonies are shown for susceptible/low-MIC M-series isolates (M1–M4; panels **A through D**) and resistant/high-MIC N-series isolates (N1–N4; panels **E through H**). Colonies showed villous mycelial growth with central green-to-yellow pigmentation, consistent with conidiogenesis and pigmented conidia under the 25°C mycelial culture condition.

The comprehensive experimental workflow adopted in this study is schematically illustrated in Fig. 1. To systematically dissect the molecular mechanisms driving azole resistance in *T. marneffei*, we employed an integrated multi-omics strategy combining transcriptomics, Data-Independent Acquisition (DIA) proteomics, and RT-qPCR validation. Clinical isolates were first documented morphologically (Fig. 2) and phenotypically stratified into resistant and susceptible cohorts based on MIC profiles before downstream omics integration.

### Fungal strains, cultivation, and identification

Eight clinical isolates of *T. marneffei* were obtained from patients with advanced AIDS complicated by talaromycosis at The Fourth People’s Hospital of Nanning. The isolates were selected from an available clinical isolate collection after antifungal susceptibility testing to represent contrasting azole MIC phenotypes, including four resistant/high-MIC isolates and four susceptible/low-MIC isolates. Species identification was supported by morphological assessment and ITS sequencing. For morphological documentation, isolates were cultured in potato dextrose agar medium at 25°C. *T. marneffei* formed filamentous colonies composed of vegetative hyphae with a villous surface. The colony center showed green-to-yellow pigmentation, consistent with conidiogenesis and the production of pigmented conidia. Representative mycelial-phase colony morphologies of the susceptible M-series isolates (M1–M4) and resistant N-series isolates (N1–N4) are shown in Fig. 2. To obtain the mycelial phase used for multi-omics analysis, conidial suspensions were inoculated into Sabouraud dextrose broth and incubated at 25°C for 3 to 10 days. All experiments were conducted using three independent biological replicates unless otherwise specified.

### Antifungal susceptibility testing and grouping

The susceptibility profiles of the isolates to VOC, FLC, and other antifungal agents were determined using the broth microdilution method in accordance with the Clinical and Laboratory Standards Institute (CLSI) M38-A2 (for filamentous fungi) and M27-A3 (for yeast) guidelines. Briefly, yeast-phase cells were collected, dispersed, and adjusted to a concentration of 2–5 × 10⁶ colony-forming unit (CFU)/mL. This suspension was diluted 1:100 in RPMI 1640 medium buffered with MOPS (pH 7.0) to achieve a 2× working concentration of 2–5 × 10⁴ CFU/mL (final inoculum size: 1–2.5 × 10⁴ CFU/mL). Serial dilutions of antifungal agents were prepared in 96-well plates. The concentration ranges were 4–0.008 μg/mL for VOC, posaconazole (POS), itraconazole (ITC), and isavuconazole (ISA); 128–0.25 μg/mL for FLC; and 16–0.031 μg/mL for amphotericin B (AmB).

Plates were incubated at 25°C (for mycelial phase) and 37°C (for yeast phase) for 72 h. The minimum-inhibitory concentration (MIC) was defined as the lowest concentration causing significant growth inhibition compared with the drug-free control. Assay quality control included *Candida krusei* ATCC 6258 and *Candida parapsilosis* ATCC 22019. And the standard strain FRR2161 of *T. marneffei* was used as the reference strain for drug susceptibility testing. Based on the MIC profiles, the isolates were categorized into a resistant group (*n* = 4, high MICs) and a susceptible group (*n* = 4, low MICs).

### Sample collection for omics analysis

Upon confirmation of the mycelial growth phase, fungal pellets from both the resistant and susceptible groups were harvested by centrifugation at 5,000 × *g* for 10 min at 4°C. The pellets were washed twice with ice-cold sterile PBS to remove the residual medium. For subsequent omics analysis, the samples were ground individually in liquid nitrogen to a fine powder and stored at −80°C until extraction. Both transcriptomic and proteomic analyses were performed on mycelial-phase fungal material collected under the basal culture condition before antifungal exposure. The two omics layers were generated from matched phenotypic groups and comparable mycelial-phase preparations and were interpreted as parallel basal profiling data sets rather than drug-induced time-course measurements.

### Transcriptomic profiling

Total RNA was extracted from the ground tissue using the TRIzol reagent (Invitrogen, USA) following the manufacturer’s instructions. Genomic DNA was removed using DNase I (TaKara). RNA quality was determined using a 2100 Bioanalyzer (Agilent Technologies, USA) and quantified using an ND-2000 (NanoDrop Technologies). High-quality RNA samples (OD260/280 = 1.8–2.2, OD260/230 ≥ 2.0, RIN ≥ 6.5, >10 µg) were used to construct the sequencing libraries. RNA-seq transcriptome libraries were prepared using 1 μg of total RNA with the VAHTS Universal V10 RNA-seq Library Prep Kit (MGI). Messenger RNA was isolated via polyA selection using oligo(dT) beads, fragmented, and subjected to cDNA synthesis, end repair, and adapter ligation. Libraries were size-selected for 200–300 bp cDNA target fragments on 2% Low Range Ultra Agarose, followed by PCR amplification using Phusion DNA polymerase (NEB) for 15 cycles. After quantification by TBS380 Picogreen (Invitrogen), the final libraries were sequenced on the MGI-T7 next-generation sequencing platform (Shanghai BIOZERON Biotech. Co., Ltd.) using the PE150 mode.

Raw paired-end reads were trimmed and quality-controlled using Trimmomatic (v0.36) with parameters SLIDINGWINDOW:4:15 MINLEN:75. Adapter sequences and reads containing >10% N bases or low-quality bases (Q < 20) were removed. Clean reads were aligned to the *T. marneffei* reference genome using Hisat2 with default parameters. The quality of the alignment was assessed using Qualimap (v2.2.1), and gene read counts were calculated using featureCounts (Subread package). Differential expression analysis was performed using the R statistical package edgeR. Differentially expressed genes (DEGs) were identified based on a logarithmic fold change (log2FC) >2 and a False Discovery Rate (FDR) < 0.05.

### Proteomic profiling

The frozen sample powder was lysed with SDT buffer (containing 100 mM NaCl and 1/100 volume of DTT), followed by 5 min of ultrasonication on ice. The lysate was incubated at 95°C for 8–15 min, cooled on ice, and centrifuged at 12,000 × *g* for 15 min at 4°C. The supernatant was alkylated with sufficient iodoacetamide (IAM, Sigma) for 1 h at room temperature in the dark. Proteins were precipitated by mixing with 4 volumes of precooled acetone at −20°C for at least 2 h. After centrifugation and washing with cold acetone, the pellet was dissolved in Dissolution Buffer (DB buffer: 6 M urea, 100 mM TEAB, pH 8.5). Protein concentration was measured using the Bradford protein quantification kit (Biognosys). For digestion, trypsin (Promega) and 100 mM TEAB buffer were added to the protein solution, mixed, and digested at 37°C for 4 h, followed by overnight digestion with additional trypsin. The digest was acidified with formic acid, centrifuged, and the supernatant was desalted using a C18 column. The eluents were lyophilized and stored for analysis.

The lyophilized powder was dissolved in 10 μL of solvent A (100% water and 0.1% formic acid). Liquid chromatography was performed on a Vanquish Neo UHPLC system (Thermo Fisher Scientific). The sample was loaded onto a C18 pre-column (174500, Thermo) and separated on a C18 analytical column (PepMap Neo UHPLC, 150 μm × 15 cm, 2 μm) at 50°C. The elution gradient used mobile phase B (80% acetonitrile and 0.1% formic acid) increasing from 2.5% to 35% over a linear gradient, followed by a wash at 99% B. Mass spectrometry was performed using an Orbitrap Astral mass spectrometer (Thermo Fisher Scientific) in the Data-Independent Acquisition (DIA) mode. The ion spray voltage was set to 2.0 kV, and the ion transfer tube temperature was 290°C. The full MS scan range was 380–980 *m/z* with a resolution of 240,000 (MS1). The DIA window size was 2 Th (300 windows), with a secondary *m/z* acquisition range of 150–2,000 and an Astral resolution of 80,000.

Raw DIA data files were analyzed using DIA-NN software. The search parameters included automatic correction of mass deviations, fixed modification of cysteine alkylation, and variable modification of N-terminal methionine excision (max two missed cleavages). Peptide Spectrum Matches (PSMs) were filtered at a 1% FDR. Protein quantification and differential expression analysis were conducted based on the output from DIA-NN.

### RT-qPCR validation

Candidate genes retained from the transcriptomic and integrated multi-omics screening were evaluated by RT-qPCR in the resistant and susceptible isolate groups. Total RNA was extracted using the RNAprep Pure polysaccharide and polyphenol plant total RNA extraction kit, and reverse transcription was performed using HiScript II Q RT SuperMix for qPCR with gDNA wiper. RT-qPCR was performed on a Roche LightCycler 480 real-time PCR system using ChamQ Universal SYBR qPCR Master Mix. Act was used as the internal reference gene. Each reaction contained SYBR qPCR master mix, forward and reverse primers, cDNA template, and nuclease-free water in a final reaction volume of 20 uL. Each sample was measured with technical replicates. The relative expression was calculated using the 2^-DeltaDeltaCt method, and resistant (R) and susceptible (S) groups were compared at the group level. *P* values below 0.05 were considered statistically significant.

### Functional enrichment and integrated network analysis

Functional annotation and enrichment analysis were conducted to interpret the biological significance of DEGs and differentially expressed proteins (DEPs). For the transcriptome, Gene Ontology (GO) enrichment and KEGG pathway analysis were carried out using Goatools and KOBAS, respectively. Terms and pathways with a Bonferroni-corrected *P*-value < 0.05 were considered significantly enriched. For visualization of transcriptomic signatures, DEGs were grouped into five biologically interpretable modules that were most directly connected to the resistance phenotype and RT-qPCR validation: efflux transport, cell wall and membrane remodeling, stress/redox/detoxification, signal transduction and regulatory adaptation, and basal/mitochondrial metabolism. Genes were selected from the DEG table according to the functional annotation and relevance to the resistance-associated modules. For the proteome, functional analysis was performed using InterProScan against non-redundant protein databases, followed by module-level visualization of resistance-associated DEP patterns. To integrate the data sets, the filtered outputs from edgeR (transcriptome) and DIA-NN (proteome) were subjected to joint pathway analysis and supervised machine learning (DIABLO framework), as described in the *Statistical analysis* section, facilitating the identification of core biomarkers and regulatory networks associated with azole resistance.

### Supervised machine learning for biomarker discovery (DIABLO)

To assess the concordance between transcriptomic and proteomic changes, matched genes/proteins were subjected to a nine-quadrant analysis. The x-axis represents transcriptomic log2 fold change, and the y-axis represents proteomic log2 fold change for the resistant group relative to the susceptible group. Each axis was divided into three intervals: log2FC < -1,-1 ≤ log2 FC ≤1, and log2FC > 1, generating nine regulatory quadrants. The central quadrant (Q5) represents features without marked changes at either omics level, whereas Q3 and Q7 represent concordant upregulation and concordant downregulation, respectively. Pearson’s correlation coefficients were calculated globally and separately within each quadrant.

### Statistical analysis

To identify robust molecular signatures driving resistance, we employed Data Integration Analysis for Biomarker discovery (DIABLO) using the mixOmics package (v6.18.1). A multi-block sparse Partial Least Squares Discriminant Analysis (sPLS-DA) model was constructed to integrate the transcriptomic and proteomic data sets. This supervised multivariate framework was utilized to select highly discriminative features that maximize the correlation between omics blocks and effectively separate the resistant and susceptible phenotypic groups. For model optimization, the optimal number of variables was determined using 10-fold cross-validation. Discriminant features were subsequently identified based on Variable Importance in Projection (VIP) scores, where key features were defined by high loading weights contributing to the group separation. To visualize the inter-omics connectivity, a relevance network was constructed to display robust correlations between the selected genes and proteins, utilizing a correlation coefficient threshold of >0.7.

## RESULTS

### Distinct antifungal susceptibility profiles stratify *T. marneffei* clinical isolates

**Fig 3:**
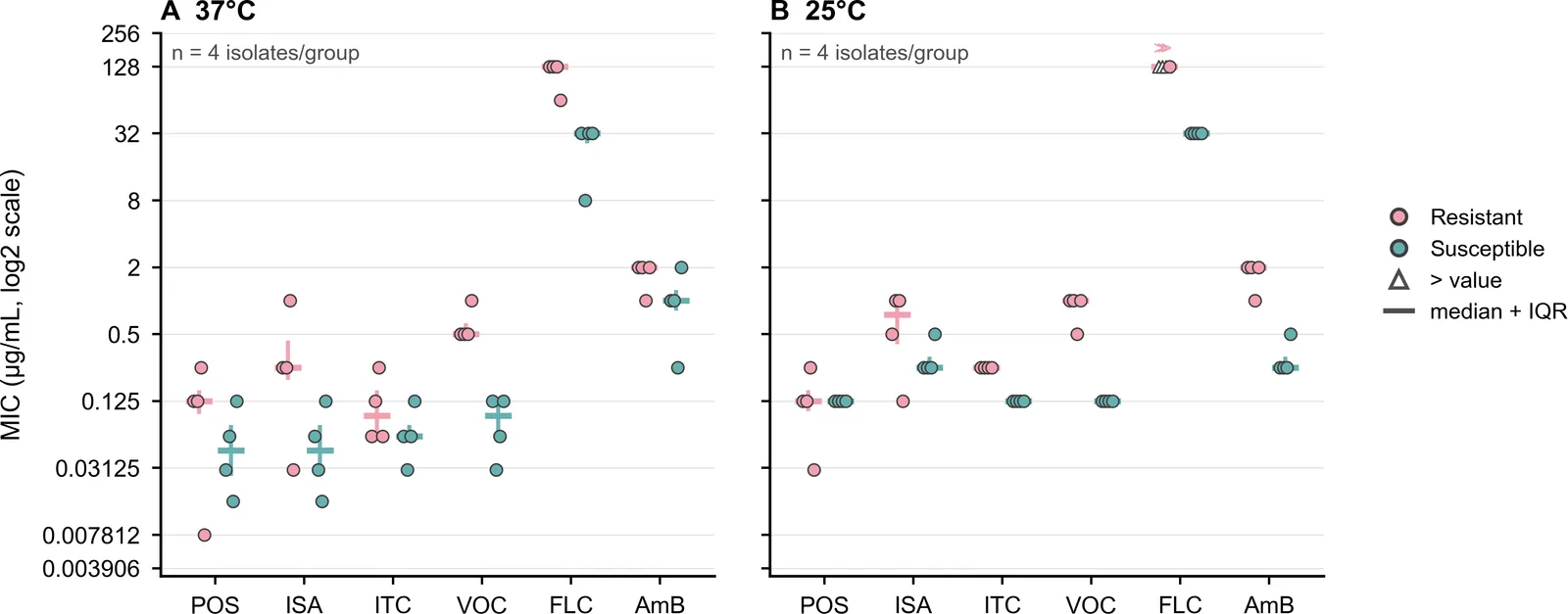
Antifungal susceptibility profiles of resistant and susceptible *T. marneffei* clinical isolates. MICs of six antifungal agents were determined for four resistant N-series isolates and four susceptible M-series isolates at (A) 37°C (yeast-phase growth) and (B) 25°C (mycelial-phase growth). Each point represents one clinical isolate, and the summary bars show the group median and interquartile range. Both panels use the same log2-scaled MIC axis. Values exceeding the highest concentration tested were plotted at the assay upper limit and marked with the “>” symbol. POS, posaconazole; ISA, isavuconazole; ITC, itraconazole; VOC, voriconazole; FLC, fluconazole; AmB, amphotericin B. n = 4 isolates per group.

To establish an integrated foundation for multi-omics analysis, we first characterized the susceptibility landscapes of eight *T. marneffei* clinical isolates against six antifungal agents. The resistant group (N-series) showed markedly higher MICs to voriconazole and fluconazole than the susceptible group (M-series), supporting the MIC-based phenotypic stratification used for downstream omics analyses (Fig. 3). MICs measured at 25°C and 37°C are displayed on the same log2 scale to allow direct comparison between temperature conditions. The strongest group separation was observed for voriconazole and fluconazole, whereas other antifungal agents showed more limited or temperature-dependent differences. These isolate-level MIC patterns provided the biological context for investigating constitutive molecular features associated with azole resistance. The standard strain FRR2161 yielded MICs of 0.0078, 0.0156, 0.03, 0.06, 2, and 1 µg/mL at 37°C and 0.0078, 0.0156, 0.03, 0.06, 2, and 0.5 µg/mL at 25°C for POS, ISA, ITC, VOC, FLC, and AmB, respectively.

### Transcriptomic dissection reveals five resistance-associated functional modules

**Fig 4:**
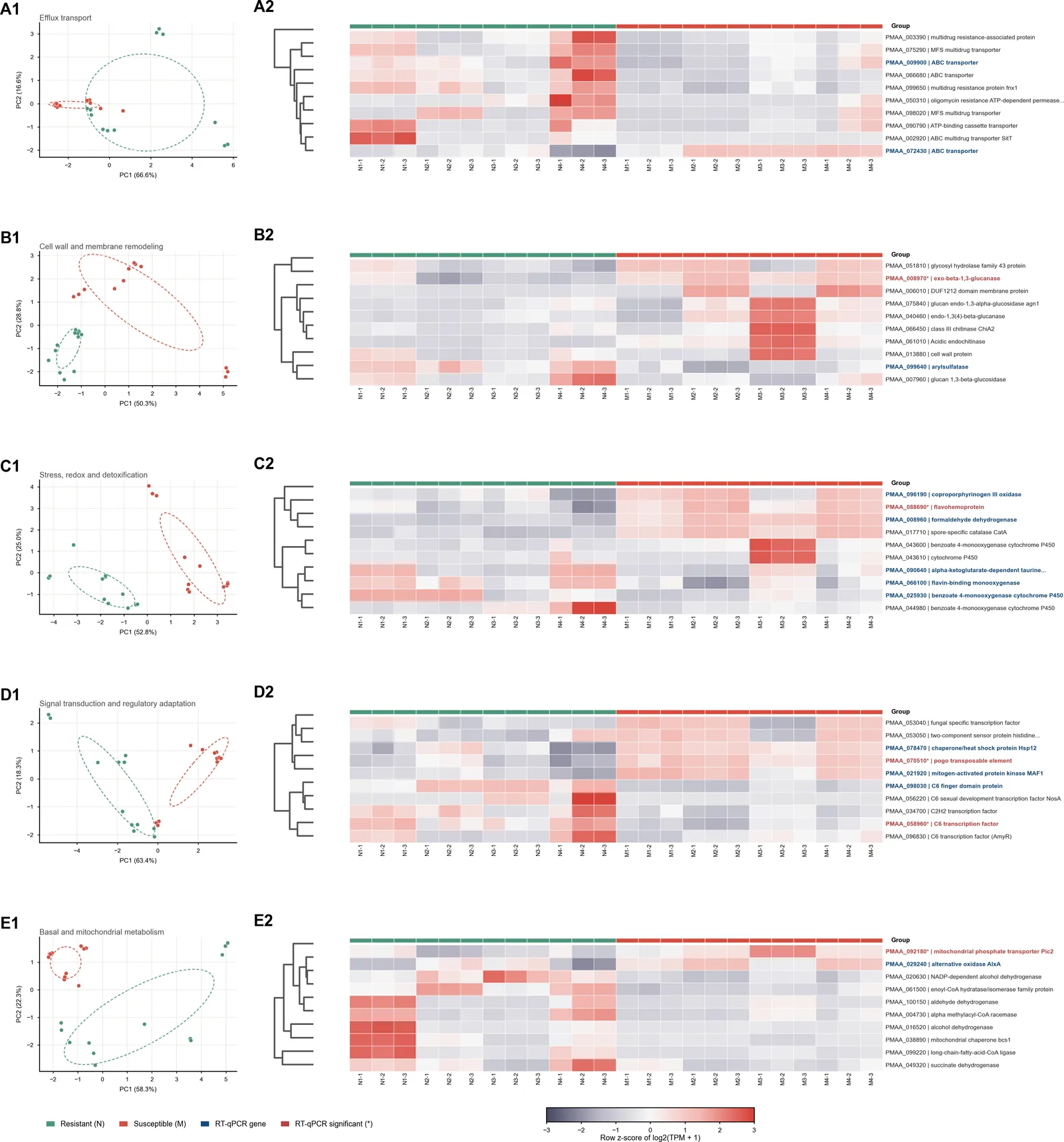
Transcriptomic dissection of five resistance-associated functional modules. For each module, the left panel shows PCA based on scaled log2(TPM + 1) expression values of module genes, and the right panel shows hierarchical clustering heatmaps based on row-wise Z-score-transformed log2(TPM + 1) values. (**A**) Efflux transport. (**B**) Cell wall and membrane remodeling. (**C**) Stress, redox, and detoxification. (**D**) Signal transduction and regulatory adaptation. (**E**) Basal and mitochondrial metabolism. Gene selection was based on DEG annotation and resistance-related functional relevance. Differential expression was defined using the transcriptomic DEG criteria described in the *Methods*. Genes included in the RT-qPCR validation panel are highlighted, and RT-qPCR-significant genes are marked with an asterisk.

To clarify the transcriptomic architecture underlying azole resistance, we reorganized the DEG-based visualization into five functional modules that were most directly connected to the resistance phenotype and RT-qPCR validation: efflux transport, cell wall and membrane remodeling, stress/redox/detoxification, signal transduction and regulatory adaptation, and basal/mitochondrial metabolism (Fig. 4). PCA and hierarchical clustering showed that resistant and susceptible isolates were separated to different extents across these modules, indicating that the resistant phenotype was associated with coordinated but module-specific transcriptional remodeling.

#### Efflux transport and cell envelope remodeling

The efflux transport module showed higher expression of multiple ABC and MFS transporter-related genes in resistant isolates, supporting enhanced drug transport capacity as a major component of the resistance-associated transcriptomic profile (Fig. 4A). In parallel, the cell wall and membrane remodeling module revealed differential expression of glucanase, chitinase, and membrane-associated genes, suggesting that altered cell-envelope organization may accompany the resistant phenotype (Fig. 4B). One RT-qPCR-tested cell wall gene, exo-beta-1,3-glucanase, was significantly different between the two groups, supporting the relevance of this module in the RT-qPCR expression panel.

#### Stress, redox, and detoxification responses

The stress/redox/detoxification module captured divergent basal redox strategies between resistant and susceptible isolates (Fig. 4C). Cytochrome P450-related enzymes, flavin-containing oxidoreductases, catalase-associated features, and other detoxification-related genes contributed to the separation between groups. Among the RT-qPCR-tested genes, flavohemoprotein showed significant differential expression, supporting the involvement of redox-associated genes in the resistance-linked transcriptomic signature.

#### Regulatory adaptation and mitochondrial metabolism

The signal transduction and regulatory adaptation module included fungal transcription factors and signaling genes that may coordinate the basal resistant state (Fig. 4D). A C6 transcription factor showed significant differential expression by RT-qPCR. The basal and mitochondrial metabolism module further highlighted genes related to respiratory and mitochondrial functions (Fig. 4E), including mitochondrial phosphate transporter Pic2, which was also significant in the RT-qPCR validation data set. These results suggest that the resistant phenotype is linked not only to transport-related changes but also to regulatory and metabolic reprogramming.

### Proteomic validation confirms translational execution of resistance traits

**Fig 5:**
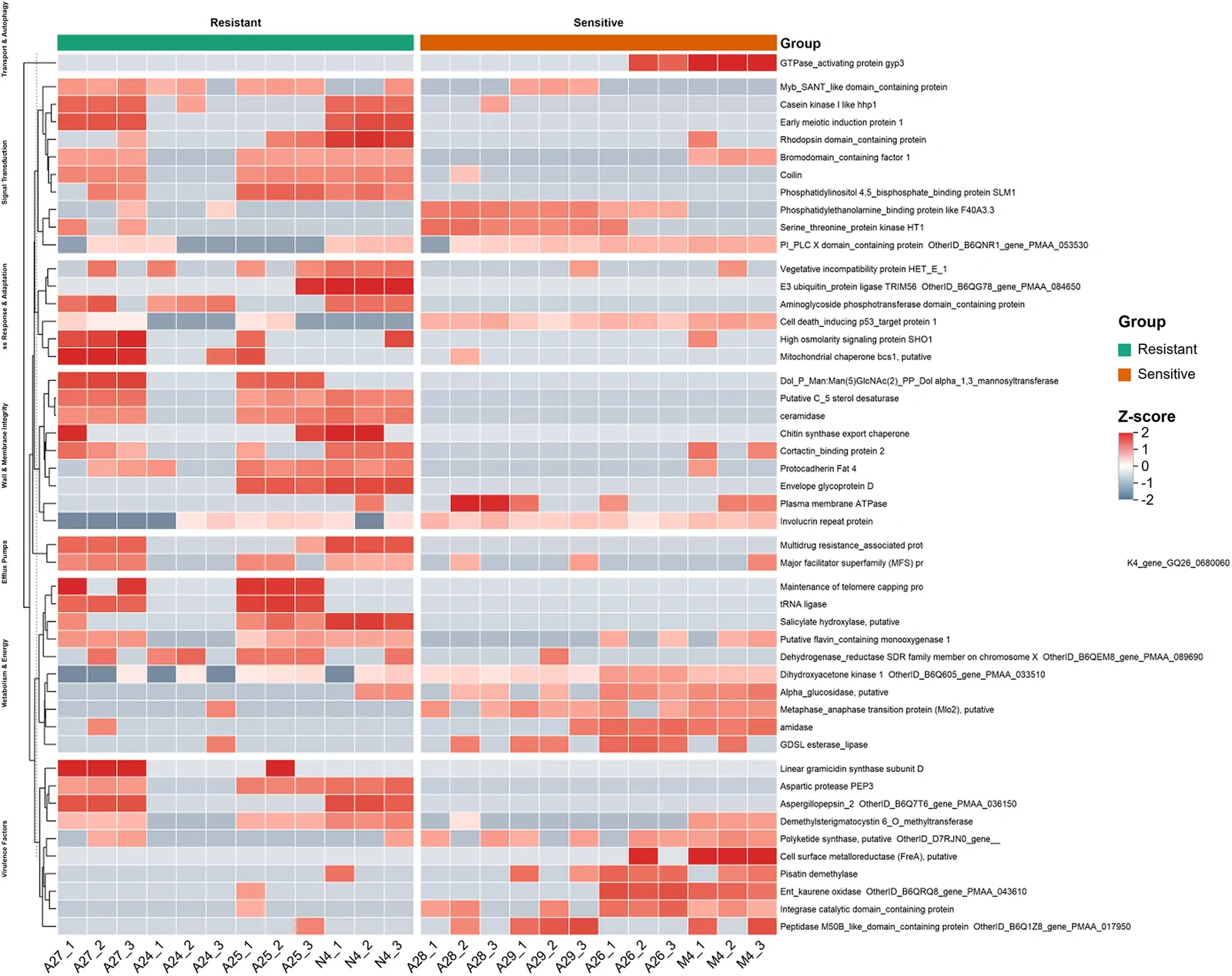
Proteomic profiling of resistance-associated modules. Hierarchical clustering heatmap of differentially expressed proteins (DEPs) between resistant and susceptible isolates. Rows represent DEPs, and columns represent proteomic samples. Color intensity indicates row-wise Z-score-transformed protein abundance. Group annotation and the Z-score scale are displayed separately from the heatmap to improve readability. The heatmap summarizes protein-level patterns across resistance-related modules, including transport, cell-envelope remodeling, stress/signaling, and metabolic or virulence-associated effectors.

Targeted DIA proteomic analysis further defined the protein-level execution of resistance-associated traits (Fig. 5). Differentially expressed proteins clustered by susceptibility phenotype and highlighted transporter-associated proteins, cell-envelope remodeling factors, stress/signaling components, and metabolic or virulence-associated effectors. These protein-level patterns were consistent with the transcriptomic modules and indicated selective translation of resistance-associated processes.

#### Defense effectors

Proteomic profiling identified resistance-associated transport and cell-envelope proteins, including Multidrug Resistance-Associated Protein 1 (MDR1) and chitin synthase export chaperones, as multi-omics-prioritized candidates consistent with transporter-associated defense and cell-wall remodeling. Because these proteins were not part of the focused RT-qPCR-supported panel, they were interpreted as proteomic support signals rather than members of the focused RT-qPCR panel.

#### Stress and virulence

The enrichment of high-osmolarity signaling protein SHO1 and Casein Kinase I suggested a heightened state of environmental surveillance. Linear gramicidin synthase and the aspartic protease PEP3 were retained as proteomic candidate signals linking resistance-associated molecular remodeling with potential virulence-related traits, but they were not treated as members of the focused RT-qPCR panel.

### Functional enrichment analysis delineates a hierarchical resistance logic

**Fig 6:**
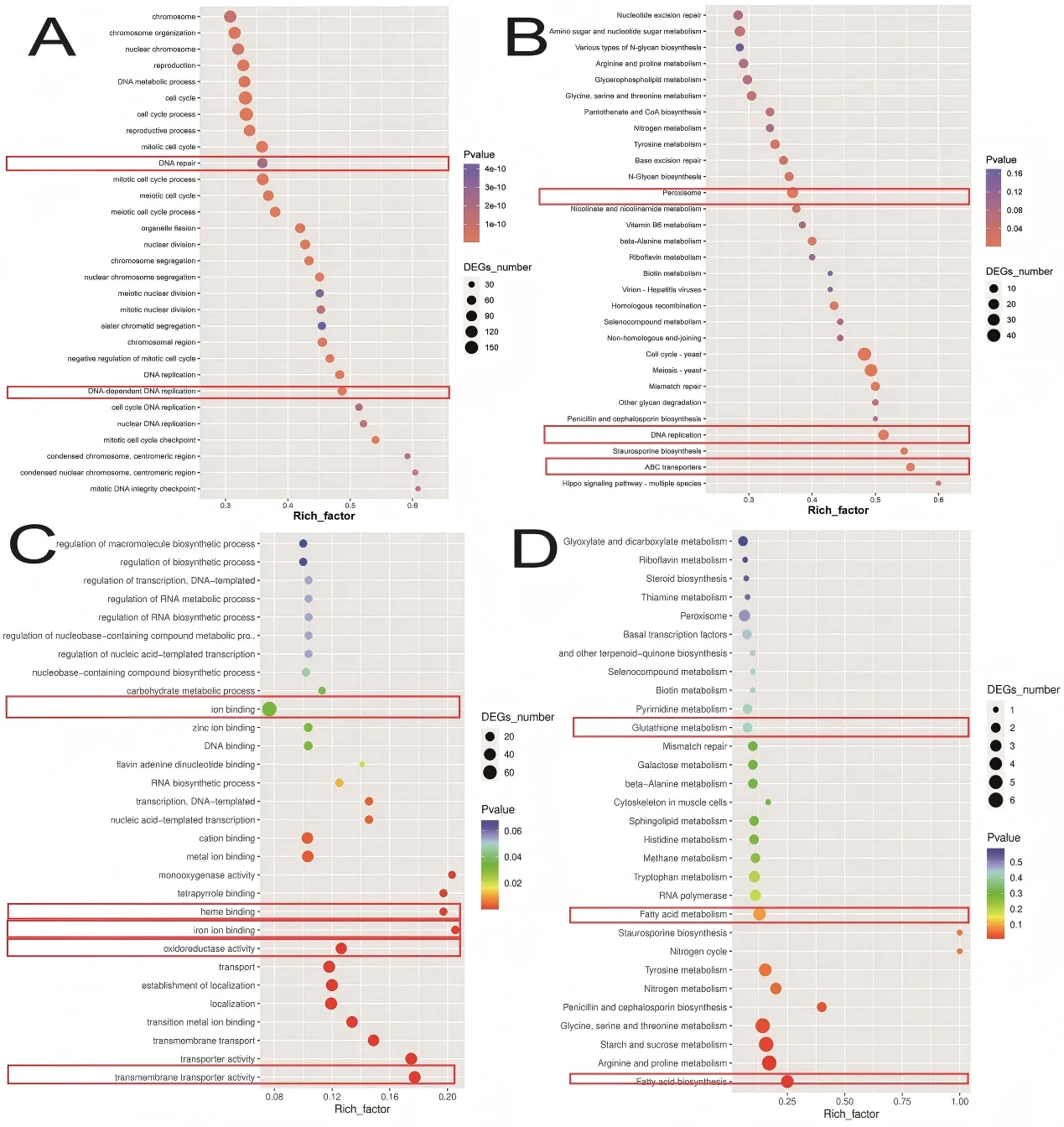
Functional enrichment of transcriptomic and proteomic changes in resistant versus susceptible T. marneffei isolates. (**A**) Gene Ontology (GO) terms enriched among transcriptomic differentially expressed genes (DEGs). (**B**) Kyoto Encyclopedia of Genes and Genomes (KEGG) pathways enriched among transcriptomic DEGs. (**C**) GO terms enriched among proteomic differentially expressed proteins (DEPs). (**D**) KEGG pathways enriched among proteomic DEPs. The x-axis shows the rich factor, defined as the proportion of genes or proteins assigned to a term or pathway that were differentially expressed. Bubble size represents the number of DEGs in panels A and B or DEPs in panels C and D, and color represents the enrichment P value, with warmer colors indicating smaller P values. Red boxes identify selected terms and pathways emphasized in the analysis. Transcriptomic enrichment was dominated by cell-cycle and genome-maintenance functions, whereas proteomic enrichment emphasized ion-binding, oxidoreductase, and transporter functions, together with glutathione, lipid, fatty-acid, and secondary metabolism.

GO and KEGG enrichment analyses were used to summarize the functional consequences of the DEG and DEP data sets (Fig. 6). The transcriptomic landscape was dominated by processes related to cell proliferation and genome maintenance, such as “Cell cycle,” “DNA replication,” and “Mismatch repair” (Fig. 6). This suggests that the transcriptional machinery primarily modulates cell cycle dynamics to support survival under stress. In contrast, the proteomic data emphasized metabolic execution. Significant enrichment was found in “Transmembrane transporter activity,” “Fatty acid biosynthesis,” and “Secondary metabolism” (Fig. 6). Collectively, these enrichment patterns support a hierarchical view in which broad transcriptional remodeling is coupled to selective protein-level execution of transport and metabolic functions.

### Integrated multi-omics analysis reveals “Transcriptional buffering” vs “Execution modules”

**Fig 7:**
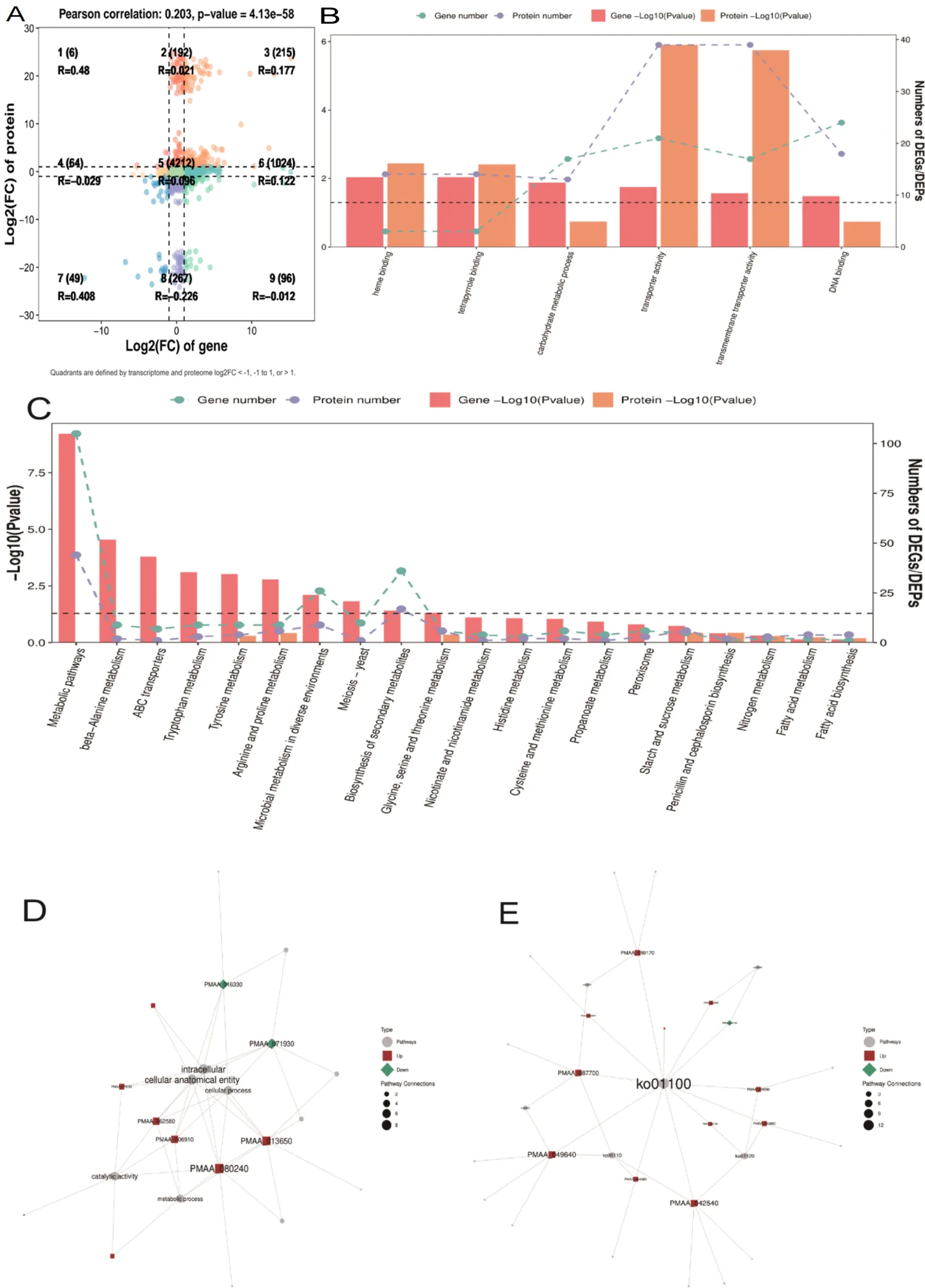
Integrated multi-omics analysis of resistant and susceptible *T. marneffei* isolates. (**A**) Nine-quadrant analysis comparing transcriptomic log2 fold change (x-axis) and proteomic log2 fold change (y-axis) for matched genes/proteins. Positive fold-change values indicate higher abundance in resistant isolates, whereas negative values indicate higher abundance in susceptible isolates. Dashed lines indicate the thresholds log2FC = −1 and log2FC = 1 on both axes, defining nine regulatory quadrants. The number in each quadrant indicates the number of matched features, and Pearson correlation coefficients are shown globally and for each quadrant. (**B**) GO enrichment comparison between transcriptomic and proteomic data sets. (**C**) KEGG pathway enrichment analysis. (**D**) Gene-concept network showing associations between differentially expressed targets and enriched GO terms. (**E**) Gene-pathway network highlighting metabolic pathway connections and representative resistance-associated enzymes.

To evaluate the degree of concordance between mRNA abundance and protein abundance, we matched transcriptomic and proteomic fold changes and performed a nine-quadrant analysis (Fig. 7A). The global Pearson correlation was low but significant (R = 0.203, *P* = 4.39 × 10^−58; *n* = 6,125 matched features), indicating that the two omics layers were only partially coupled at the global level. Quadrant-specific analysis separated concordant and discordant transcript-protein features. Concordant upregulated features were assigned to Q3 (*n* = 215, R = 0.177, *P* = 0.009), whereas features with increased transcript abundance but unchanged protein abundance were assigned to Q6 (*n* = 1,024, R = 0.122, *P* = 9.10 × 10^−5). These patterns indicate that a subset of resistance-associated genes is translated into protein-level execution modules, while a larger set is subject to post-transcriptional buffering or delayed protein-level deployment.

### DIABLO-based machine learning prioritizes multi-omics candidate signals

**Fig 8:**
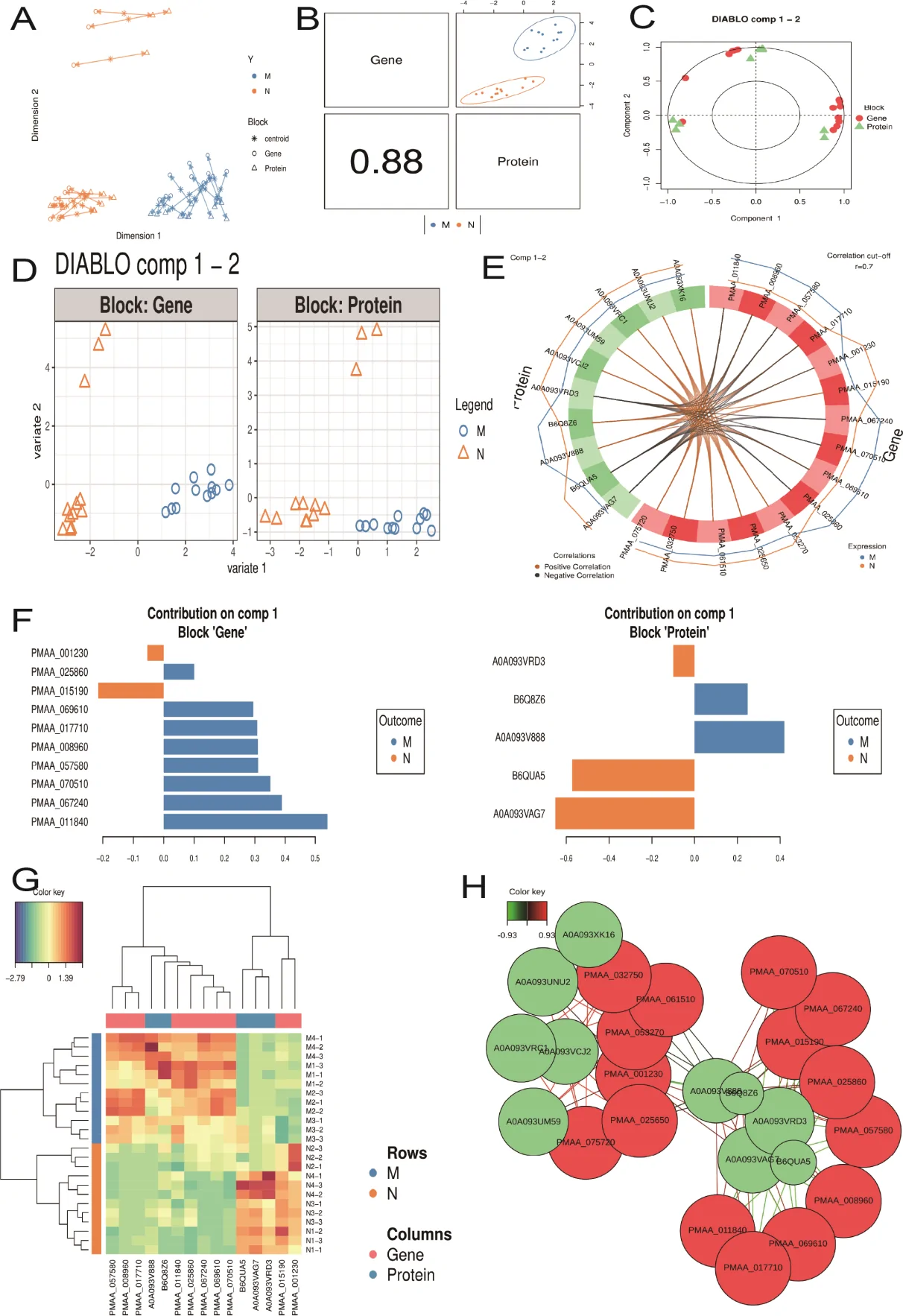
Supervised multi-omics integration using DIABLO prioritizes candidate signals associated with azole-response phenotypes. (**A**) Arrow plot visualizing the projection of samples into the integrated latent space. Resistant and susceptible groups are separated. (**B**) Scatter plot matrix showing the correlation between transcriptomic and proteomic latent components. (**C**) Correlation circle plot displaying covariation of selected features. (**D**) Individual sample plots showing the discriminative contribution of omics blocks. (**E**) Circos plot visualizing the correlations between discriminatory genes and proteins. (**F**) Loading plots ranking multi-omics-prioritized candidate features. (**G**) Clustered image map showing candidate-feature abundance patterns across isolates. (**H**) Relevance network highlighting stress-related and regulatory candidate modules. DIABLO-selected features highlight regulatory, RNA-processing, stress-response, and genome-instability signals associated with azole-response phenotypes.

Data Integration Analysis for Biomarker discovery (DIABLO) was used to prioritize discriminatory molecular features across transcriptomic and proteomic layers. The model separated resistant and susceptible isolates in the integrated latent space and identified features contributing to group discrimination (Fig. 8A through D).The resistant-associated pattern included features linked to RNA processing and fungal transcriptional regulation, whereas the susceptible-associated pattern included stress and genome instability-related signals such as CatA-associated oxidative stress and Pogo transposable-element activation (Fig. 8E through H). Together, these DIABLO-selected features outlined RNA-processing, regulatory, stress-response, and genome-instability axes that complemented the RT-qPCR-supported expression panel.

### RT-qPCR validation supports a 17-gene resistance-associated expression panel

RT-qPCR analysis was performed for a 17-gene expression panel in resistant and susceptible isolates. The panel covered four functional categories: transport/cell-wall processes, redox-detoxification, mitochondrial metabolism, and stress/regulatory adaptation. All 17 genes yielded detectable RT-qPCR signals and were retained for expression-based resistance profiling. Five genes showed statistically significant R/S expression differences. The C6 transcription factor showed higher relative expression in resistant isolates, whereas exo-beta-1,3-glucanase, Pogo transposable element, flavohemoprotein, and mitochondrial phosphate transporter Pic2 showed lower relative expression in resistant isolates than in susceptible isolates. These statistically supported genes connected the RT-qPCR layer to cell-wall remodeling, regulatory adaptation, redox homeostasis, genome stress-associated signaling, and mitochondrial transport.

The other 12 panel members, namely, ABC transporter, arylsulfatase, formaldehyde dehydrogenase, benzoate 4-monooxygenase cytochrome P450, flavin-binding monooxygenase, alpha-ketoglutarate-dependent taurine dioxygenase, coproporphyrinogen III oxidase, alternative oxidase AlxA, C6 finger-domain protein, mitogen-activated protein kinase MAF1, heat shock protein Hsp12, and a second stress-regulatory candidate, were detectable and showed module-consistent directional trends without reaching the *P* < 0.05 significance threshold. Overall, RT-qPCR analysis supported a multi-gene resistance-associated expression panel spanning transport/cell-wall remodeling, redox-detoxification, mitochondrial metabolism, and stress- regulatory adaptation.

**Fig 9:**
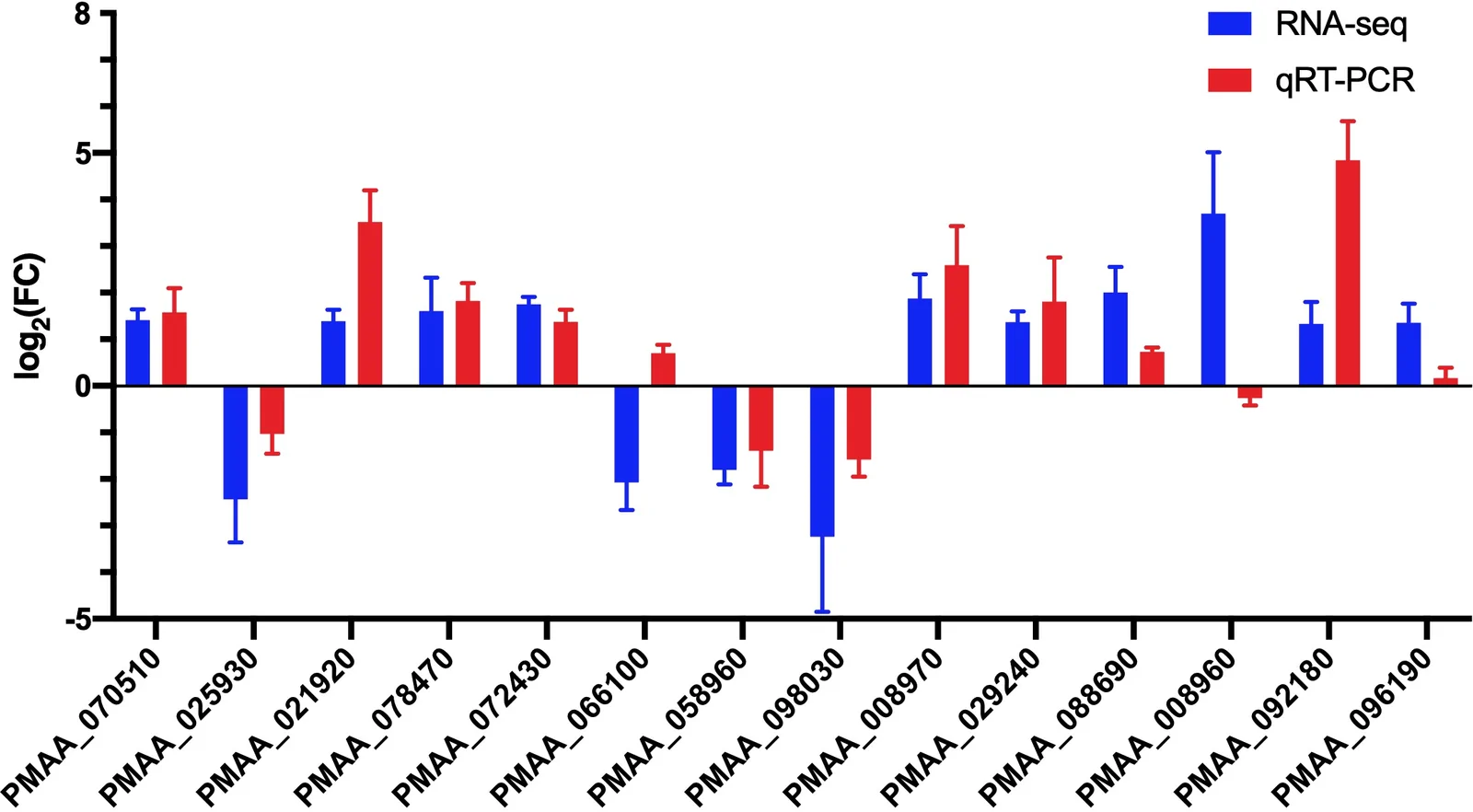
RNA-seq and qRT-PCR fold-change comparison for selected resistance-associated genes. Blue bars indicate RNA-seq-derived log2 fold-change estimates, and red bars indicate qRT-PCR-derived log2 fold-change estimates. Error bars show measurement variation. The comparison provides expression-level support for the qRT-PCR validation layer and shows broadly concordant or module-consistent patterns for the displayed candidate genes, while preserving gene-specific differences in effect size between platforms.

Direct comparison of RNA-seq and qRT-PCR fold-change estimates further supported the expression-level consistency of the selected validation genes (Fig. 9). Most displayed genes showed concordant direction or module-consistent expression patterns between the two platforms, while differences in fold-change magnitude were retained as expected quantitative variation between RNA-seq and qRT-PCR measurements.

## DISCUSSION

Our integrated transcriptomic and proteomic analysis of *T. marneffei* clinical isolates indicates that azole resistance is associated with a constitutive, multitiered defense configuration rather than a single-gene effect. By comparing the basal molecular profiles of resistant (N-series) and susceptible (M-series) strains under mycelial conditions (25°C), we identified a coordinated resistance-associated program involving cell wall/transport, redox-detoxification, mitochondrial/energy metabolism, and regulatory adaptation. Vesicular trafficking, RNA-processing, and virulence- associated signals further extended this architecture, linking the expression panel to membrane delivery, post-transcriptional control, and pathogenicity-associated metabolism.

### Coordinated upregulation of efflux transporters and vesicular trafficking machinery

A primary mechanism of azole resistance in fungi involves the reduction of intracellular drug accumulation through transporter-mediated efflux (25, 26). In *T. marneffei*, the transport layer involved ABC transporter-associated transcriptional changes together with proteomic enrichment of transporter-related proteins. Multidrug Resistance-Associated Protein 1 (MDR1), SitT, MFS transporter signals, and the RT-qPCR-supported ABC transporter collectively indicate reinforced transport capacity in high-MIC isolates.

Intracellular transport and vesicular trafficking also contributed to the transport/cell-envelope module, particularly in isolate N4. The exocyst component Sec8 and COPI coatomer proteins connect resistance-associated transport to vesicle tethering and membrane-protein delivery. Because efflux activity depends on correct localization of membrane transporters, enhanced vesicular delivery could help sustain transporter function; inhibition of vesicular trafficking increases fluconazole susceptibility in fungal models (27).

The cell-wall component of the model was supported by transcriptomic and RT-qPCR evidence for cell-wall remodeling candidates, including exo-beta-1,3-glucanase and arylsulfatase, together with proteomic signals involving chitin synthase export chaperones. Chitin synthases are integral to cell wall synthesis and maintenance, and their chaperoning may reflect altered cell-wall remodeling dynamics under the resistant phenotype (28, 29). Together, these transport and cell-wall signals support a coordinated candidate module that could influence drug entry, cell-envelope integrity, and transporter deployment, while requiring functional perturbation to establish causality.

### Divergent strategies in basal redox homeostasis and detoxification

Azole antifungals, in addition to inhibiting ergosterol synthesis, often induce oxidative stress through the accumulation of reactive oxygen species (ROS) and toxic sterol intermediates (30, 31). Our results indicate that resistant and susceptible strains exhibit distinct basal metabolic profiles regarding redox homeostasis.

The resistant-associated redox-detoxification layer was supported by RT-qPCR panel members including flavohemoprotein, formaldehyde dehydrogenase, benzoate 4-monooxygenase cytochrome P450, flavin-binding monooxygenase, and taurine dioxygenase. Flavohemoprotein showed statistically significant RT-qPCR differences, while the remaining genes were detectable and trend-supporting within the same module. These signals are consistent with a basal detoxification and redox-buffering state that may help resistant isolates manage azole-associated oxidative and xenobiotic stress (30–32).

CatA-associated oxidative-stress and autophagy-related features were enriched in the susceptible phenotype, highlighting a distinct basal stress state. Hydrogen peroxide exposure induces fungal oxidative-stress responses, including antioxidant enzyme systems such as catalases (33), and the co-occurrence of CatA-associated signals with autophagy-related markers suggests that susceptible isolates may rely more strongly on stress-response and turnover pathways than resistant isolates.

### Constitutive expression of virulence factors and bioenergetic support

It is often hypothesized that the evolution of antimicrobial resistance incurs a fitness cost, potentially leading to reduced virulence or growth rates due to the energetic burden of resistance mechanisms (34). However, our data from *T. marneffei* clinical isolates do not support a trade-off between resistance and virulence under basal conditions, aligning with reports in other pathogenic fungi where certain azole-resistant clinical isolates exhibit no detectable fitness cost or even enhanced competitiveness (35). Transcriptomic and proteomic analyses also revealed resistance-associated signals involving secondary-metabolite and virulence-related proteins, including polyketide synthase and non-ribosomal peptide synthetase candidates, linear gramicidin synthase, and the aspartic protease PEP3 (Fig. 4 and Fig. 5). These features connect resistance-associated physiology with secretion, secondary metabolism, and host-interaction pathways, indicating that high-MIC isolates maintain defense and pathogenicity-associated functions under basal growth.

**Fig 10:**
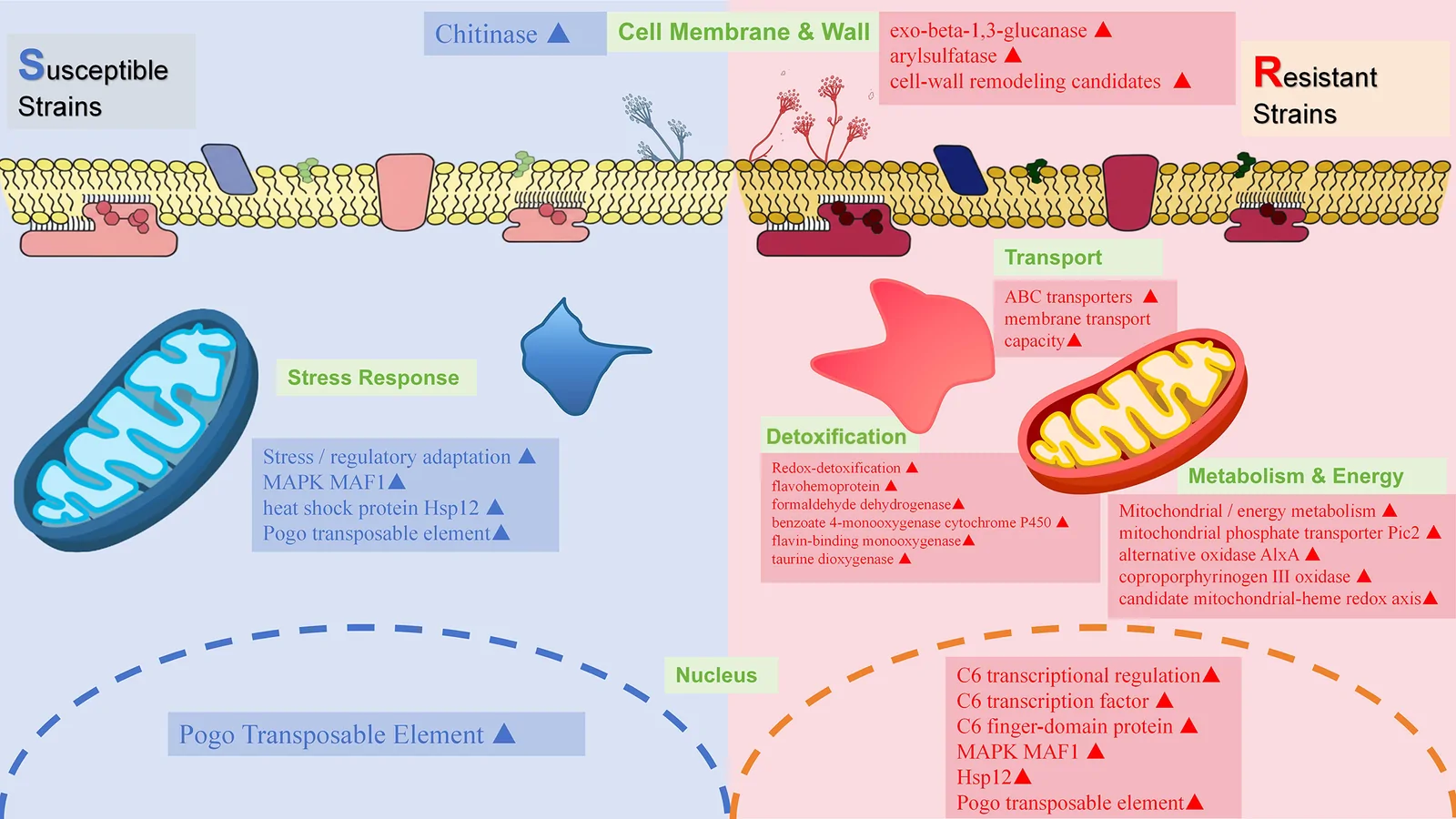
Schematic model of the molecular characteristics of azole-resistant *Talaromyces marneffei*. The model highlights four resistance-associated modules, namely, cell wall/transport, redox-detoxification, mitochondrial/energy metabolism, and regulatory adaptation. Asterisks indicate genes with statistically significant RT-qPCR differences; unmarked genes show detectable module-consistent expression trends. The 17-gene panel integrates with transcriptomic and proteomic signatures to define a coordinated resistance-associated molecular network in high-MIC isolates.

Mitochondrial and energy-metabolism components were prominent within the RT-qPCR-supported panel. Mitochondrial phosphate transporter Pic2 showed significant R/S expression differences, and alternative oxidase AlxA provided a trend-supporting respiratory signal. Together, these findings link the expression panel to phosphate handling, respiratory flexibility, and energy metabolism, processes that can influence stress tolerance and antifungal adaptation in pathogenic fungi (36, 37). These patterns define a bioenergetic module in which mitochondrial metabolism operates alongside transport and redox-detoxification processes in high-MIC isolates. The four-module model in Fig. 10 places mitochondrial energy handling as a coordinated component of the resistance-associated state rather than as an isolated virulence signal.

The RT-qPCR-supported panel further links mitochondrial metabolism to iron- and heme-associated redox regulation. Alternative oxidase AlxA and mitochondrial phosphate transporter Pic2 connect the panel to respiratory flexibility and mitochondrial energy handling, whereas coproporphyrinogen III oxidase and flavohemoprotein connect this layer to heme-associated redox metabolism. This relationship is consistent with *T. marneffei* studies showing that *AcuM* and *AcuK* regulate genes involved in mitochondrial carbon metabolism, oxidative-stress response, iron-containing proteins, and heme/iron-binding functions and that acuK contributes to iron acquisition and gluconeogenic adaptation (38, 39). Intracellular iron chelation can also impair fungal mitochondrial respiration in *Candida species* (40). Together, these observations support a mitochondrial-iron/heme redox axis in which respiratory activity, iron availability, and heme-related detoxification jointly shape the high-MIC phenotype.

### Post-transcriptional regulation and hierarchical control

The integration of transcriptomic and proteomic data sets revealed a relatively low global correlation (Pearson R = 0.203) between mRNA and protein levels (Fig. 7A). This discrepancy, a common observation in multi-omics studies attributable to extensive post-transcriptional regulation (41), is particularly evident in the large cluster of genes with upregulated mRNA but unchanged protein levels (Quadrant 6 in Fig. 7A), pointing to active post-transcriptional regulatory mechanisms.

This pattern is consistent with a “transcriptional priming” or mRNA reserve strategy, whereby resistant strains constitutively transcribe and accumulate mRNAs encoding stress-response factors under basal conditions, maintaining them in a translationally repressed state often through sequestration in stress granules or processing bodies (42, 43). Such storage enables cells to bypass delays associated with chromatin remodeling and *de novo* transcription upon encountering environmental stress, thereby facilitating a rapid proteomic response. Conversely, the “Execution Module” (Quadrant 3), characterized by coordinated upregulation of both mRNA and protein levels, was enriched for transporter activity and metabolic enzymes. This pattern supports a hierarchical regulatory logic: the transcriptome appears broadly pre-programmed to anticipate diverse stresses (encompassing cell cycle and DNA repair pathways, as revealed by GO analysis), whereas the proteome is selectively tuned for immediate execution of transport and metabolic functions.

The specific enrichment of Zn(2)-C6 fungal-type transcription factors, including NosA—which plays a key role in stress tolerance and developmental responses in *Aspergillus fumigatus* (44)—and AmyR, which regulates amylolytic gene expression in *Aspergillus oryzae* (45), suggests that fungal transcriptional regulators may coordinate the constitutive transcriptional program. These transcription factors are likely responsible for driving basal transporter and metabolic gene expression, thereby contributing to the intrinsic resistant phenotype.

### Molecular markers of resistance: RNA processing versus genomic instability

The supervised data integration analysis (DIABLO) provided additional candidate signals distinguishing resistant and susceptible phenotypes. Coilin (A0A093VAG7) was identified as a discriminatory feature for the resistant group (Fig. 8F). Coilin is a scaffold protein of Cajal bodies, nuclear sub-organelles involved in small nuclear ribonucleoprotein biogenesis and spliceosome assembly (46, 47). Coilin was prioritized by DIABLO and links the resistant phenotype to RNA-processing architecture. Efficient RNA processing and splicing can influence fungal growth, stress adaptation, and antifungal responses, and recent work demonstrates functional links between Cajal body or spliceosomal components and fungal morphogenesis or growth (46–49).

Within the stress-adaptation layer, the Pogo transposable element showed significant differences in RT-qPCR, supporting its role as a candidate marker in the regulatory-adaptation module. In fungi, transposable elements can act as stress-adaptive genomic elements and promote genome instability under stress (50). Sge1- and CatA-associated DIABLO features further connect this module to transcriptional control, oxidative stress, and genome-stability responses.

### Conclusion

In conclusion, our integrated multi-omics analysis characterized the basal molecular landscape of azole-resistant *Talaromyces marneffei* clinical isolates (Fig. 10). The analysis supports a 17-gene RT-qPCR-supported candidate panel associated with cell wall/transport, redox-detoxification, mitochondrial/energy metabolism, and regulatory adaptation. Resistant isolates showed coordinated basal remodeling of transport capacity, cell-envelope processes, detoxification, mitochondrial bioenergetics, and stress-regulatory circuits. These findings identify candidate molecular markers and mechanism-linked pathways for azole-response stratification in *T. marneffei*.

## Data Availability

The RNA-seq read data have been deposited in the NCBI Sequence Read Archive (SRA) under BioProject accession number PRJNA1457906 and SRA study accession number SRP700848. The 24 paired-end RNA-seq libraries are available under run accession numbers SRR38626017 to SRR38626040, with BioSample accession numbers SAMN57481783 to SAMN57481806. Proteomic data have been deposited in iProX under accession number IPX0017012000.
